# Following the Central Dogma: A Review of Fluoroquinolone Resistance Mechanisms in Aquatic Pathogenic Bacteria

**DOI:** 10.3390/ijms27219603

**Published:** 2026-10-27

**Authors:** Lei Zhu, Cangle Dai, Mengcao Wang, Xinyi Zhu, Jing Ma, Yihan Zhang, Yong Zhao, Huan Li, Zhaohuan Zhang

**Affiliations:** 1 College of Food Science and Technology, Shanghai Ocean University, 999# Hu Cheng Huan Road, Shanghai 201306, China; d250400156@st.shou.edu.cn (L.Z.); m250451390@st.shou.edu.cn (C.D.); m250401303@st.shou.edu.cn (M.W.); m250401183@st.shou.edu.cn (X.Z.); m250451414@st.shou.edu.cn (J.M.); m250401279@st.shou.edu.cn (Y.Z.); yzhao@shou.edu.cn (Y.Z.); h-li@shou.edu.cn (H.L.); 2 International Research Center for Food and Health, Shanghai Ocean University, 999# Hu Cheng Huan Road, Shanghai 201306, China; 3 Laboratory of Quality & Safety Risk Assessment for Aquatic Products on Storage and Preservation (Shanghai), Ministry of Agriculture and Rural Affairs, 999# Hu Cheng Huan Road, Shanghai 201306, China; 4 Shanghai Engineering Research Center of Aquatic-Product Processing & Preservation, 999# Hu Cheng Huan Road, Shanghai 201306, China

**Keywords:** aquatic pathogenic bacteria, fluoroquinolones, resistance mechanisms, central dogma, target-site mutation

## Abstract

Fluoroquinolone resistance in aquatic bacterial pathogens is a growing concern in aquaculture worldwide. Multidrug-resistant isolates are increasingly reported across major farming regions, and the potential transmission of resistance genes to humans through the food chain and environmental exposure has attracted attention beyond aquaculture. Most existing reviews discuss individual resistance mechanisms, such as target-site mutations, plasmid-mediated quinolone resistance (PMQR), efflux pumps, and altered membrane permeability, as discrete topics, with limited consideration of their interrelationships. This review takes a different approach. Using the central dogma of molecular biology as an organizing framework, it organizes fluoroquinolone resistance into four interconnected levels: gene, transcription, protein, and phenotype. Gene-level resistance involves point mutations in the quinolone resistance-determining region (QRDR) and the acquisition of PMQR genes. Transcriptional responses include coordinated upregulation of efflux pumps and downregulation of porins, which together reduce intracellular drug accumulation. Protein-level mechanisms include altered drug targets, target protection by Qnr proteins, drug efflux, and enzymatic modification of the drug. At the phenotypic level, PMQR-mediated low-level resistance provides a survival window during which subsequent QRDR mutations accumulate, whereas efflux pump overexpression confers higher-level resistance. Biofilm formation and metabolic reprogramming further stabilize the resistance phenotype at the population level. This framework highlights how resistance mechanisms interact across different molecular and cellular levels, rather than functioning independently or following a strictly linear sequence. Key gaps remain in understanding the regulatory roles of non-coding RNAs, the structural biology of PMQR proteins, and the use of multi-omics approaches to clarify regulation across these levels. Further research is needed to address these gaps.

## 1. Introduction

Fluoroquinolones are a landmark in the history of antibacterial drug development [1,2]. Since the introduction of the first fluorinated quinolone in the 1980s, this class has become a cornerstone of antibacterial therapy in humans and animals, owing to its broad antibacterial spectrum, potent bactericidal activity, excellent tissue penetration, and favorable pharmacokinetic profile [3,4,5,6]. In aquaculture, fluoroquinolones such as enrofloxacin, ciprofloxacin, and norfloxacin have been used to manage major bacterial diseases, including those caused by *Aeromonas* spp., *Vibrio* spp., and *Edwardsiella* spp. [7]. However, their approval and permitted use vary substantially among jurisdictions, and “wide use” here refers to observed practice and surveillance findings rather than uniform authorization. Long-term, large-scale use of these agents where permitted has nonetheless made bacterial resistance a growing problem [8].

Misuse of fluoroquinolones leads to several adverse outcomes. After medicated feeding, the fraction of the administered fluoroquinolone that is absorbed varies widely with compound, species, dose, temperature, and administration route; even when absorption is relatively efficient, a substantial proportion of the parent compound and its active metabolites can still be excreted via feces and uneaten feed into aquaculture water, where they accumulate in sediments and exert persistent sublethal selection [9,10]. This long-term selective pressure at sublethal concentrations drives the emergence and spread of fluoroquinolone resistance in aquatic pathogens [11,12]. In recent years, national and international resistance surveillance data show that resistance to enrofloxacin and ciprofloxacin among major aquatic pathogens, including *Aeromonas hydrophila*, *Vibrio parahaemolyticus*, and *Edwardsiella tarda*, has reached high levels in some farming regions [13,14,15], and the isolation rate of multidrug-resistant strains has risen annually [16,17,18,19]. More concerning, resistance genes can be horizontally transferred between bacterial species and across ecological niches via mobile genetic elements such as plasmids, integrons, and transposons [20], and this documented mobility makes the spread of resistance beyond aquaculture a plausible risk whose occurrence has not yet been directly established [21,22].

Understanding the mechanisms of fluoroquinolone resistance in aquatic pathogenic bacteria is essential for resistance surveillance, rational clinical antibiotic use, and the development of novel antibacterial strategies. Decades of research have shifted the focus from phenotypic descriptions to molecular mechanisms [23,24]. Fluoroquinolone resistance is now known to involve multiple mechanisms. Target-site mutations often contribute to high-level resistance, although their effects vary among bacterial species and genetic backgrounds [24]; plasmid-mediated quinolone resistance (PMQR) genes spread horizontally within bacterial populations [20]; and overexpression of efflux pump systems together with altered membrane permeability further reduces intracellular drug accumulation [25,26]. These mechanisms are not mutually exclusive, and strains often carry more than one [27]. Reviewing these mechanisms and their interactions can inform strategies to slow the emergence of resistance.

Several reviews have summarized fluoroquinolone resistance mechanisms [25,26,27,28], but most treat them as separate categories, addressing target-site mutations, plasmid-mediated resistance, efflux pumps, and membrane permeability changes as discrete topics [16,25]. Although this classification is clear, it tends to obscure the interactions among resistance mechanisms at different molecular and cellular levels. The central dogma of molecular biology provides a useful conceptual framework for organizing these mechanisms from genetic determinants through transcription and protein function to resistance-associated phenotypes. However, this framework does not imply a strictly linear or universal sequence of resistance development, as different mechanisms may arise independently, coexist, or interact. This review therefore adopts a four-level framework of gene, transcription, protein, and phenotype as its organizing principle to integrate current knowledge on fluoroquinolone resistance mechanisms in aquatic pathogenic bacteria. After reviewing the application and resistance status of fluoroquinolones, we examine the molecular mechanisms at each level, summarize current knowledge gaps, and propose future research directions to inform resistance surveillance, antimicrobial stewardship, and control strategies in aquaculture.

## 2. Fluoroquinolones in Aquaculture

### 2.1. Discovery and Action Mechanism

The fundamental difference between quinolones and fluoroquinolones lies in the parent nucleus and the modification of key substituents. Quinolones share the 4-quinolone core; early agents based on this core underwent only limited structural modification, which resulted in a narrow antibacterial spectrum and suboptimal pharmacokinetic properties [18]. Nalidixic acid, the first representative drug of this class, was discovered in 1962 and was used mainly to treat urinary tract infections caused by Gram-negative bacteria [19]. Introducing a fluorine atom at the C-6 position of the parent nucleus markedly enhanced antibacterial activity, tissue penetration, and bioavailability, giving rise to the fluoroquinolones and marking a pivotal milestone in the development of this antibiotic class [21,22].

Based on antibacterial spectrum and clinical characteristics, fluoroquinolones are generally divided into four generations. The first generation, represented by nalidixic acid, has a narrow spectrum [29]. The second generation, represented by ciprofloxacin, enrofloxacin, and norfloxacin, has an expanded spectrum covering most Gram-negative bacteria, including *Pseudomonas aeruginosa*, and some Gram-positive bacteria [30]. The third generation, represented by levofloxacin, shows further enhanced activity against Gram-positive bacteria [31]. The fourth generation, exemplified by moxifloxacin, extends coverage to anaerobes [18]. This progression has been achieved mainly through structural optimization at positions C-6, C-7, and N-1 of the quinolone core.

**Figure 1:**
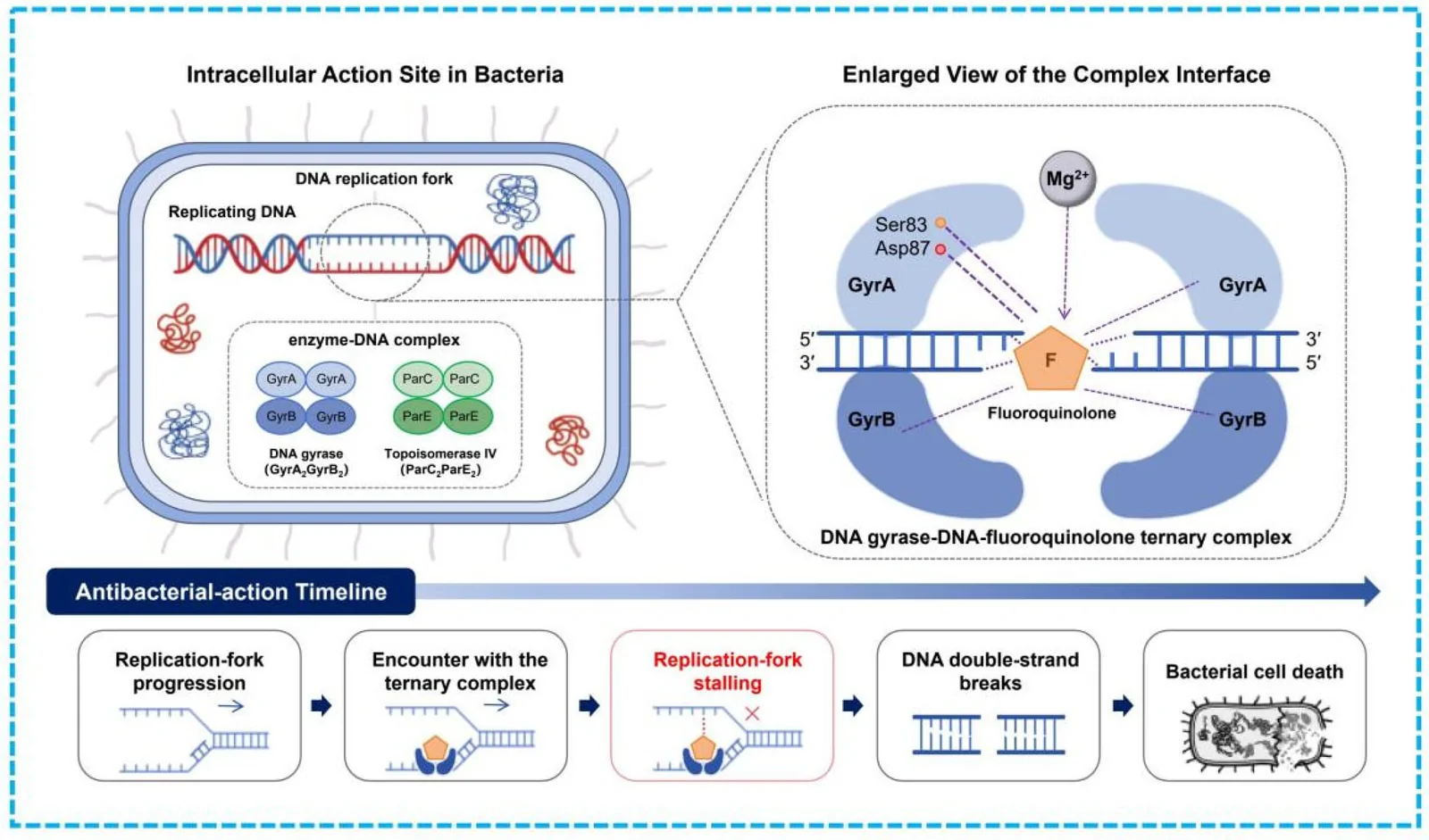
Schematic diagram of the mechanism of action of fluoroquinolones.

Fluoroquinolone antibiotics act primarily by specifically inhibiting bacterial type II topoisomerases, namely DNA gyrase (topoisomerase II, composed of GyrA_2_GyrB_2_) and topoisomerase IV (composed of ParC_2_ParE_2_) [21]. Both enzymes are essential for bacterial DNA replication, transcription, and chromosome segregation: DNA gyrase introduces negative supercoils, whereas topoisomerase IV decatenates daughter chromosomes [16]. Fluoroquinolone molecules bind reversibly at the interface of the enzyme-DNA complex, intercalating into the broken DNA strands to form a stable “fluoroquinolone-topoisomerase-DNA” ternary complex [16,25]. This binding blocks religation of the DNA strands, stalls the DNA replication fork, and induces double-strand breaks, ultimately causing bacterial cell death [19]. Structural biology studies have shown that the fluorine atom and C-7 substituents enhance affinity for the enzyme-DNA complex, and that Mg^2+^ ion bridging, for instance involving Ser83 and Asp87 of GyrA, is a key molecular feature [32,33]. In Gram-negative bacteria, DNA gyrase is generally the primary target, while in Gram-positive bacteria, topoisomerase IV plays a more prominent role [25] (Figure 1).

### 2.2. Use and Residue in Aquaculture

Fluoroquinolone antibiotics have been used increasingly in aquaculture since the 1970s [34], mainly to prevent and treat bacterial diseases caused by *Aeromonas*, *Vibrio*, and *Edwardsiella* [5,35]. Under intensive, high-density farming, these agents are often administered through medicated feed or immersion baths for mass treatment and prophylaxis [27,28]. Enrofloxacin and its metabolite ciprofloxacin are currently the most widely used fluoroquinolones in aquaculture. In China, Announcement No. 2292 of the Ministry of Agriculture (2015) prohibited the use of ofloxacin and norfloxacin in food-producing animals; enrofloxacin, however, remains heavily relied upon as a prescription drug [5,6].

In recent years, several domestic monitoring studies have documented the occurrence of fluoroquinolone residues in aquaculture. Yu et al. [36] monitored 6850 shrimp, crab, and shellfish samples in Shanghai from 2021 to 2023 and reported a detection rate of 9.0% and an exceedance rate of 1.6% for enrofloxacin; freshwater shrimp were of greatest concern, with detection and exceedance rates of 18.0% and 4.1%, respectively. The same research group further tested 9321 fish samples from 2023 to 2024 and found an enrofloxacin detection rate of 45.9% and an exceedance rate of 7.5%, with residue levels significantly higher in freshwater fish than in marine fish [37]. Liu and Cheng [38] investigated small-scale farms in Anshun, Guizhou, and reported that off-label or non-compliant drug use could reach 10%, with higher residue rates in pond aquaculture than in recirculating aquaculture systems. Lin [39] screened commercially available fish in Xiamen for residues and reported a combined exceedance rate of approximately 2.21% for enrofloxacin and ciprofloxacin. These findings suggest that some farmers administer excessive doses and fail to observe withdrawal periods, resulting in drug accumulation in aquatic product tissues [40].

These practices also have environmental consequences. The proportion of administered fluoroquinolones absorbed by aquatic animals varies with the compound, species, administration route, and farming conditions [17]. Unabsorbed drug residues and excreted metabolites may enter the water column and sediments, contributing to environmental contamination. Meng et al. [6] reported high concentrations of fluoroquinolone residues, including enrofloxacin and ciprofloxacin, in both water and sediments from major aquaculture river basins in China, such as the Yangtze River and the Pearl River. Sediments have become long-term reservoirs of antibiotics and resistance genes, continuously exerting sublethal selective pressure on microorganisms in the aquatic environment. These residues promote the emergence of resistant strains and horizontal gene transfer and can enter the food chain [41]; whether this exposure leads to adverse human health effects, including gastrointestinal, hepatic, renal, or neurological toxicity, remains uncertain [42]. Although dietary exposure assessment for Shanghai residents indicated that the overall hazard quotient is far below 1, high-consumption groups may still be at risk from cumulative exposure [36,37].

### 2.3. Resistance Surveillance

Driven by the long-term selective pressure described above, resistance to fluoroquinolones in aquatic pathogenic bacteria has been widely documented across farming regions worldwide. Resistance rates vary widely by geographic area and bacterial species, from below 5% to over 90% (Table 1). This variation may partly reflect regional differences in antibiotic use intensity and management practices as well as interspecies differences in the ability to acquire target-site mutations, carry PMQR genes, and regulate efflux pump systems.

Among Gram-negative bacteria, *Aeromonas* is the genus most intensively monitored for resistance [43,44,45,46,47]. In Jiangsu Province, China, *Aeromonas* isolates from diseased fish, shrimp, and crabs showed enrofloxacin resistance rates of 4.95% to 8.58% and ciprofloxacin resistance rates of 14.87% to 16.75% [43,44]. In contrast, *Aeromonas* isolates from diseased Chinese soft-shelled turtles (*Pelodiscus sinensis*) in Hebei Province had an enrofloxacin resistance rate of 45.6% in 2023 [46]. In eastern China, *Aeromonas hydrophila* isolated from diseased crucian carp (*Carassius auratus*) and snakehead fish (*Channa argus*) showed an enrofloxacin resistance rate of 30% [47], while *Aeromonas* isolates from diseased snakehead fish in Zhejiang Province showed 22.22% resistance to both ciprofloxacin and norfloxacin [50]. These findings indicate that even within the same genus, resistance rates to the same drug vary considerably among strains from different geographic regions and host origins. Resistance rates in *Vibrio* are generally lower: *Vibrio parahaemolyticus* from retail shrimp and oysters in Guangdong showed resistance rates of only 1.19% to enrofloxacin and 4.76% to ciprofloxacin [52], and *Vibrio* isolates from farmed Hong Kong oysters (*Crassostrea hongkongensis*) in Guangxi showed an average resistance rate of 5.1% to four fluoroquinolones [45]. Notably, resistance in *Edwardsiella piscicida* from largemouth bass (*Micropterus salmoides*) farms in Guangdong is extremely severe, with resistance rates of 96.25% to ciprofloxacin and 40% to enrofloxacin [51], indicating that fluoroquinolone treatment options for this pathogen in the region are extremely limited.

Among Gram-positive bacteria, the genus *Enterococcus* shows markedly higher resistance levels than most Gram-negative bacteria [53,58]. *Enterococcus* isolates from tilapia and shrimp in Egypt showed resistance rates of 74.1% to ciprofloxacin and 72.2% to levofloxacin [53], while *Enterococcus* from retail shrimp in the United States displayed a ciprofloxacin resistance rate as high as 84.55% [58]. This disparity between Gram-positive and Gram-negative bacteria relates to the target preference of fluoroquinolones in the two groups: in Gram-positive bacteria, topoisomerase IV is the primary target, and the mutation patterns in its ParC subunit differ from those of GyrA in Gram-negative bacteria [29,59]. Surveillance data from other major aquaculture-producing countries in Asia likewise reflect varying degrees of resistance challenges: *Aeromonas* from freshwater fish in Thailand exhibited an enrofloxacin resistance rate of 21% [54]; *Vibrio* from shrimp farming environments in Bangladesh showed resistance rates of 15.7% to ciprofloxacin and 17.7% to levofloxacin [55]; mixed bacterial isolates from freshwater fish farm water in Pakistan displayed a ciprofloxacin resistance rate of 18% [56]; and *Acinetobacter baumannii* from fish, shrimp, and lobster in Iran showed a ciprofloxacin resistance rate of 45% [57].

Two patterns emerge from these data. First, the reported resistance rates of a given drug differ markedly among the surveyed bacterial populations: the resistance rate of *Edwardsiella piscicida* to ciprofloxacin was higher than that of *Vibrio parahaemolyticus* in the cited studies, and relatively high resistance rates were also reported for *Enterococcus* in some studies. These differences may partly reflect variations in antibiotic use and interspecies differences in target-site mutation preferences, the ability to acquire PMQR genes, and the regulation of efflux pump expression. Second, resistance rates for the same species in the same geographic region can vary severalfold between adjacent years [46], indicating that resistance rates are not fixed metrics but change dynamically with antibiotic use intensity, stocking density, and environmental conditions. However, direct comparisons among these studies should be interpreted with caution because of differences in sample sizes, bacterial species, sample sources, susceptibility-testing methods, and interpretive criteria. The following sections examine the molecular basis of these differences.

## 3. Gene-Level Encoding

Bacterial resistance to fluoroquinolones originates at the genomic level through DNA sequence changes that encode resistance information [25]. Such changes arise from two sources: random mutations in chromosomal genes that are subsequently enriched under drug selection [60], and the acquisition of resistance genes from external sources via mobile genetic elements [16]. Both introduce DNA sequence changes that underlie the resistance phenotype.

### 3.1. Chromosomal Mutations

**Figure 2:**
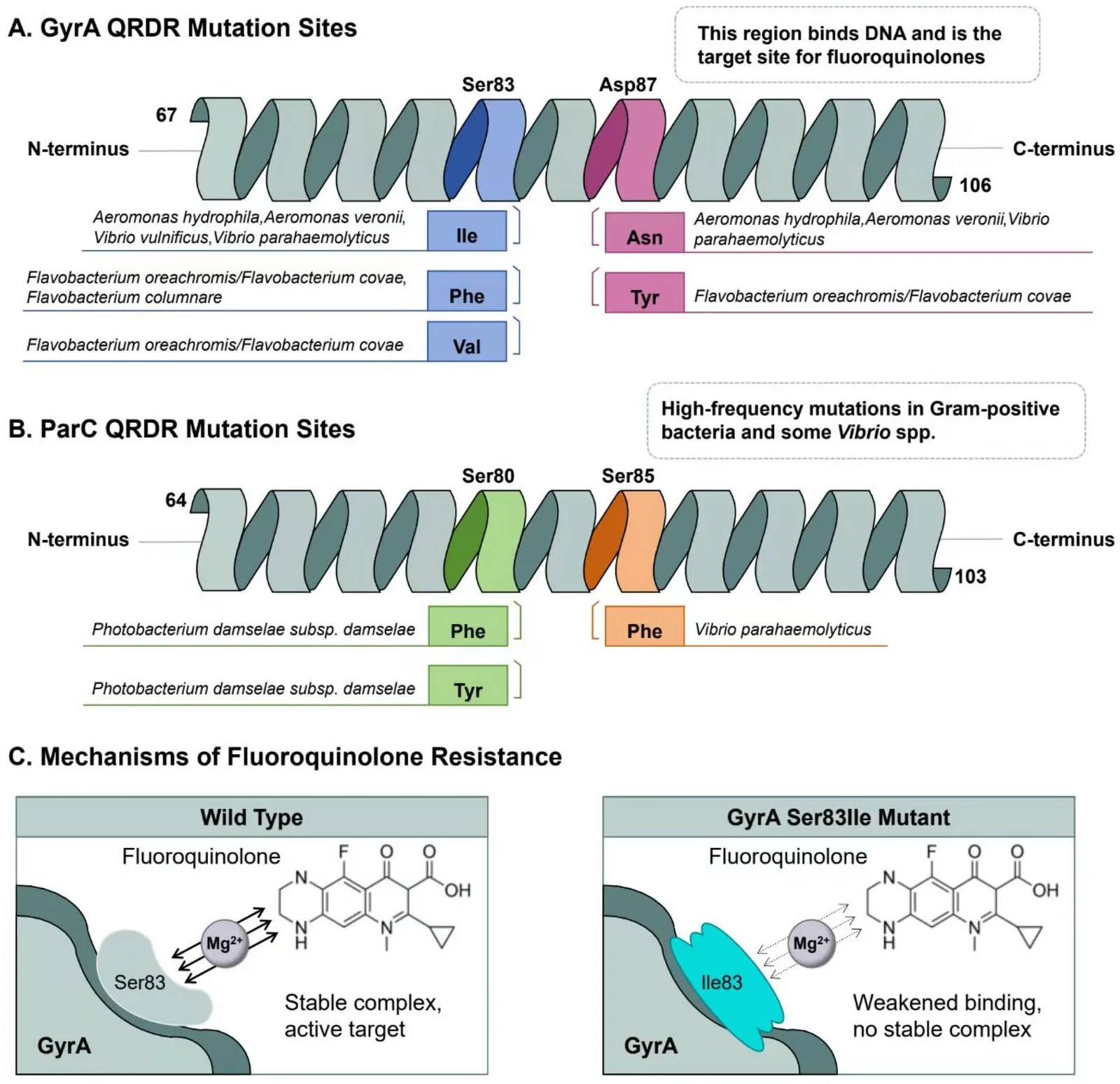
Schematic diagram of mutation sites within the quinolone resistance-determining region (QRDR).

The targets of fluoroquinolones are DNA gyrase (encoded by *gyrA* and *gyrB*) and topoisomerase IV (encoded by *parC* and *parE*) [21]. The coding sequences of these four subunit genes contain a region designated the quinolone resistance-determining region (QRDR), which encodes amino acid residues that directly participate in formation of the drug-enzyme-DNA complex [18]. Point mutations within the QRDR can alter the spatial conformation of the target enzymes, reducing their affinity for fluoroquinolone molecules and thereby allowing bacteria to maintain normal DNA replication in the presence of the drug (Figure 2) [61,62,63,64,65,66,67,68].

Among Gram-negative aquatic pathogenic bacteria, *gyrA* is the most frequently mutated gene [69,70]. The most commonly reported mutations within the QRDR are substitution of serine at position 83 (Ser83) with isoleucine, leucine, or valine, and substitution of aspartate at position 87 (Asp87) with asparagine, glycine, or tyrosine [70]. Hu et al. [61] sequenced levofloxacin-resistant *Aeromonas hydrophila* obtained after in vitro induction and found that all resistant isolates carried the *gyrA* Ser83Ile mutation, with some strains also acquiring an additional Leu92Met mutation. This indicates that Ser83 is the primary mutational hotspot in this species under fluoroquinolone selective pressure. Yang et al. [62] likewise detected *gyrA* Ser83Ile widely in *Aeromonas veronii* isolated from diseased channel catfish (*Ictalurus punctatus*) in China, and showed that this single mutation alone conferred a pronounced resistance phenotype. Nguyen et al. [63] compared genomes of *Flavobacterium* spp. causing columnaris disease in freshwater fish in Thailand and identified Ser83Phe/Val/Ala and Asp87Tyr mutations in *F. oreochromis* and *F. covae*; the latter was the first report in Thai isolates, suggesting that regional selective pressure may drive the emergence of novel mutations. Yang et al. [65] detected a high frequency of the single GyrA Ser83Ile mutation in *Photobacterium damselae* subsp. *damselae* isolated from diseased fish in eastern China. Wu et al. [67] used long-term exposure experiments that simulated sublethal levofloxacin concentrations in aquatic environments and showed that susceptible *Vibrio parahaemolyticus* could rapidly acquire key single mutations, including *gyrA* Ser83Ile, *parC* Ser85Phe, and *gyrB* Pro748Ser, within 30 days. This directly demonstrates the strong selective effect of sublethal residues on QRDR mutations.

Mutation patterns vary among bacterial species. In Gram-negative bacteria, the resistance phenotype of *Aeromonas* and *Vibrio* is driven mainly by *gyrA* mutations [61,67]. In Gram-positive bacteria such as *Streptococcus agalactiae*, by contrast, mutations in *parC* and *parE* are more prominent; Ouancharee et al. [68] found that the single mutations ParC S79F/Y and ParE H221Y in *S. agalactiae* from tilapia in Thailand are closely associated with levofloxacin resistance. This difference reflects the distinct primary targets of fluoroquinolones in the two groups: DNA gyrase is the primary target in Gram-negative bacteria, whereas topoisomerase IV serves that role in Gram-positive bacteria.

Single-target mutations usually confer only moderate resistance, with a 4- to 32-fold increase in the minimum inhibitory concentration (MIC). Under sustained drug selective pressure, however, strains carrying single mutations provide a genetic background for the subsequent accumulation of mutations across multiple genes [21]. When *gyrA* mutations occur together with additional mutations in *parC* or *parE*, both target enzymes lose drug sensitivity, and resistance can increase exponentially [71]. Zhou et al. [72] used site-directed mutagenesis to show that in a *Vibrio cholerae* background already carrying the *gyrA* S83I and *parC* S85L double mutations, introducing *gyrA* D87N raised the ciprofloxacin MIC approximately 4-fold, and subsequently introducing *parE* D420N produced a total increase of approximately 17-fold. Yang et al. [65] found that among high-level resistant *Photobacterium damselae* subsp. *damselae* strains, 94.4% (17/18) carried both GyrA Ser83Ile and ParC Ser80Phe/Tyr, and double mutants showed significantly higher resistance than single mutants. These findings indicate that combined mutations across multiple genes are a key step in the transition from low-level to high-level fluoroquinolone resistance in aquatic pathogenic bacteria.

### 3.2. Mobile Gene Acquisition

Bacteria can also acquire resistance genes from external sources through mobile genetic elements such as plasmids, integrons, and transposons, in addition to chromosomal gene mutations [20,73]. Resistance conferred by plasmid-borne determinants is termed plasmid-mediated quinolone resistance (PMQR), whereas plasmids, integrons, and transposons facilitate the dissemination of these determinants. Unlike target-site gene mutations, PMQR genes generally do not directly confer high-level resistance, typically increasing the minimum inhibitory concentration by 4- to 32-fold [23]. However, their low resistance threshold provides a survival window for bacteria in drug-containing environments, thereby creating conditions for the subsequent accumulation of target-site mutations.

Four main categories of PMQR genes are currently recognized. The *qnr* gene family encodes Qnr proteins, which belong to the pentapeptide repeat protein family and block fluoroquinolone binding to target enzymes by associating with DNA gyrase or topoisomerase IV [74], thus protecting the target. Several allelic variants of qnr genes have been reported, including *qnrA*, *qnrB*, *qnrS*, *qnrC*, *qnrD*, and *qnrVC*; among these, *qnrS* and *qnrVC* are detected more frequently in aquatic pathogenic bacteria [75]. *aac(6′)-Ib-cr* encodes an aminoglycoside acetyltransferase variant that catalyzes N-acetylation of fluoroquinolones containing a piperazine ring, such as ciprofloxacin and norfloxacin, thereby reducing their antibacterial activity [76]. *qepA* encodes a major facilitator superfamily efflux pump [77], while *oqxAB* encodes an RND-family efflux pump [78]; both lower intracellular drug concentrations through active efflux. These PMQR genes often reside on broad-host-range plasmids such as IncC, IncHI2, and IncF, and can be transferred among different bacterial species by conjugation.

PMQR genes are being detected with increasing frequency in aquaculture environments. Xu et al. [79] analyzed the genomes of non-O1/non-O139 *Vibrio cholerae* isolates from shrimp in Guangdong, China, and reported a *qnrVC* detection rate of 80.95% (17/21 strains). These genes frequently co-occurred with the target-site mutations GyrA Ser83Ile and ParC Ser85Leu, together mediating ciprofloxacin resistance or intermediate resistance. One isolate carried an IncC-type conjugative plasmid that simultaneously harbored 13 antibiotic resistance genes, indicating that PMQR genes can be co-carried with other resistance genes and have potential for horizontal dissemination. Thaotumpitak et al. [80] detected *qnrS* in 13.3% of *Aeromonas hydrophila* isolates and 65.4% of *Salmonella* spp. isolates from tilapia farming systems in Thailand; PMQR-positive strains often also carried *floR*, *sul*, and *tetA*, resulting in multidrug-resistant phenotypes. Amin et al. [81] screened *Escherichia coli* from various aquatic environments in Bangladesh and found an overall PMQR prevalence of 66%, with *qnrS* as the predominant variant (82%), followed by *aac(6′)-Ib-cr* (9%), *oqxAB* (7%), *qnrB* (6%), and *qepA* (4%). Moreover, PMQR-positive strains were significantly associated with extended-spectrum β-lactamase genes and carbapenemase genes, suggesting that PMQR genes often serve as mobile platforms that facilitate the assembly of multidrug resistance gene clusters.

Integrons also contribute importantly to the capture and expression of PMQR genes [82]. Class 1 integrons carry the integrase gene *intI1*, which captures exogenous gene cassettes through site-specific recombination and integrates them into its own locus, allowing the captured genes to be expressed under the control of the integron’s intrinsic promoter [83,84]. Thaotumpitak et al. [80] found that *intI1* and *qnrS* co-occurred at high frequency in *Aeromonas* and *Salmonella* isolates from tilapia farming sources in Thailand, and that multidrug resistance was significantly more common among integron-positive strains than among integron-negative strains. These mobile genetic elements enable PMQR genes to spread efficiently across different bacterial species and different matrices (water, sediment, fish tissues) within aquaculture environments.

## 4. Transcriptional Responses

Gene-level changes must be transcribed before they can give rise to resistance functions [85]. Transcription connects genotype to protein function [86]. At this level, bacteria regulate the transcription of specific genes to control which resistance functions are expressed and to what extent, enabling adaptive responses under drug pressure.

### 4.1. Efflux Pump Activation

**Figure 3:**
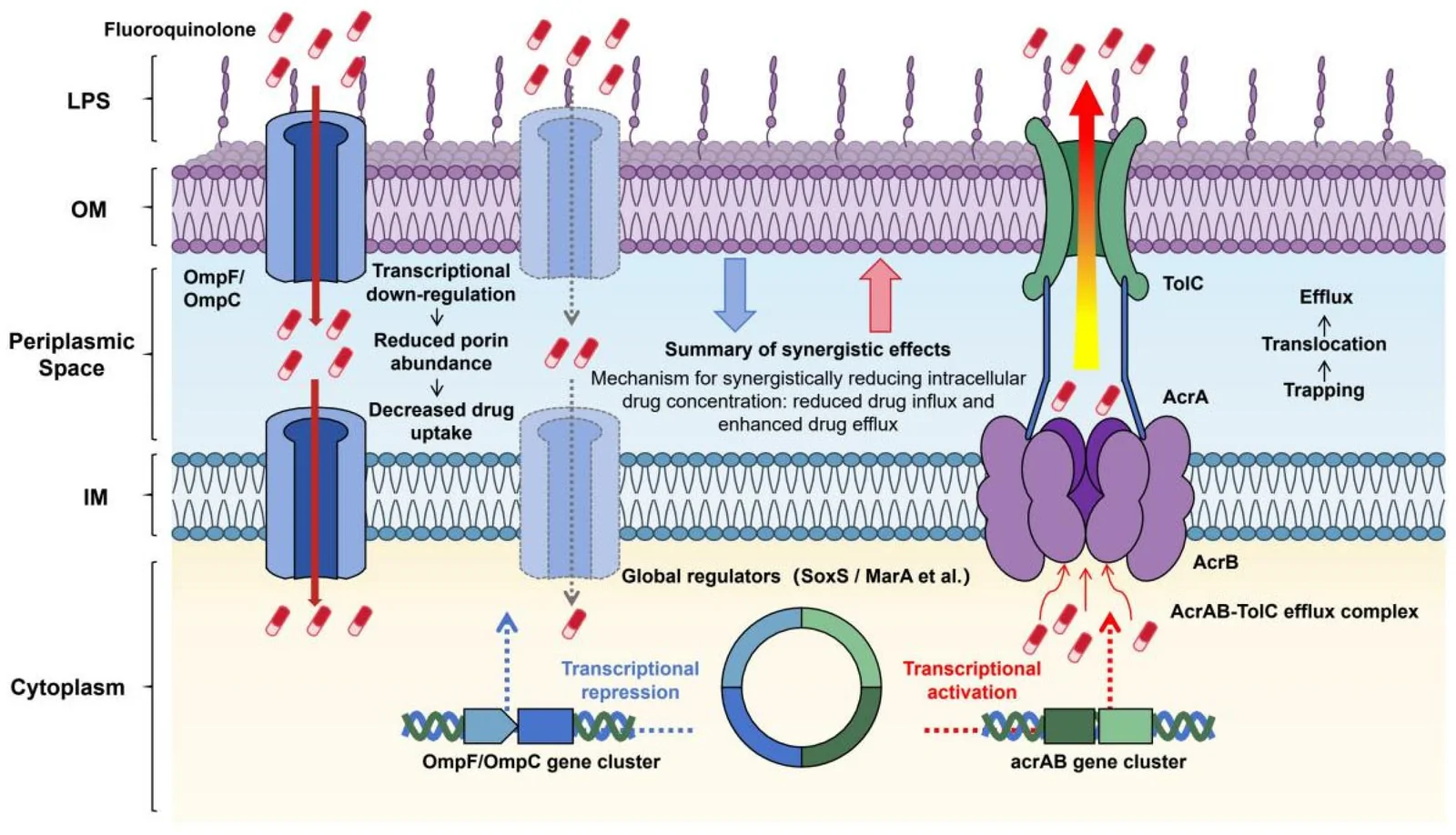
Schematic diagram of transcriptional regulation of efflux pumps and porins, and the corresponding protein execution mode.

Efflux pump systems are a core mechanism by which bacteria actively reduce intracellular drug concentrations [16,25]. In many Gram-negative aquatic pathogenic bacteria, the resistance-nodulation-division (RND) superfamily is among the major efflux systems mediating fluoroquinolone efflux [87]. RND pumps generally form tripartite complexes comprising an inner membrane transporter, a membrane fusion protein, and an outer membrane channel protein; they use the proton motive force to actively extrude fluoroquinolone molecules that enter the cytoplasm [88]. Genes encoding these components are often transcriptionally activated upon drug exposure, increasing efflux pump protein expression and thereby enhancing efflux capacity (Figure 3).

Within the genus *Aeromonas*, Wu et al. [89] generated enrofloxacin-resistant strains of *Aeromonas schubertii* by experimental induction; the MIC increased from 0.03125 μg/mL to 32 μg/mL, a 1024-fold increase. Transcriptome sequencing showed that efflux-related genes, including *acrA*, *acrB*, and the *pst* series, were significantly upregulated. After addition of the efflux pump inhibitor carbonyl cyanide m-chlorophenylhydrazone (CCCP), the MIC of the resistant strains decreased 4-fold, directly confirming that transcriptionally upregulated efflux systems contribute to maintaining high-level resistance. The strains also showed cross-resistance to flumequine and neomycin, and the resistant phenotype remained stable for 40 generations under drug-free conditions, indicating that once efflux pump genes are transcriptionally activated, the effect is genetically stable.

Within the genus *Vibrio*, Li et al. [90] performed gene knockout experiments on the VmeAB system of *Vibrio parahaemolyticus*. After knocking out *vmeB*, which encodes the inner membrane transporter, the strain showed an approximately 5-fold increase in susceptibility to enrofloxacin, along with significant reductions in biofilm formation and motility, as well as transcriptional downregulation of outer membrane-related genes (*ompA*, *ompH*, *ompN*, *ompU*, etc.) and genes encoding flagellar and fimbrial components. This finding indicates that the *vmeB* product directly mediates enrofloxacin efflux and also reinforces the resistance phenotype by regulating biofilm and outer membrane integrity, highlighting the central role of RND efflux pumps in the regulatory network of fluoroquinolone resistance in *Vibrio*.

Transcriptional activation of efflux pump genes has also been well documented in *Pseudomonas* and Enterobacteriaceae. Suresh et al. [91] isolated *Pseudomonas aeruginosa* from freshwater fish and found that 82.8% of strains exhibited efflux pump activity, with detection rates of *mexA*, *mexB*, and *oprM* (components of the MexAB-OprM system) reaching 74.2%, 77.1%, and 37.1%, respectively. Although resistance to enrofloxacin and ciprofloxacin was relatively low in this species (11.4% and 8.5%), efflux pump genes co-occurred frequently with biofilm formation genes, suggesting that the Mex system also contributes to fluoroquinolone efflux in aquatic environments. Suresh and Pillai [92] screened *Klebsiella pneumoniae* from freshwater fish farms and reported detection rates as high as 94% for *acrA*, *acrB*, and *tolC* (which constitute the AcrAB-TolC system) and 56.2% for *mdtK* (of the MATE family); all isolates displayed active efflux activity.

The transcriptional activation of efflux pump genes described above is not an isolated event but is coordinately controlled by global regulatory factors. In Gram-negative bacteria, transcriptional activators such as SoxS and MarA can simultaneously upregulate transcription of efflux pump genes such as *acrAB* and repress expression of the porin gene *ompF*, producing a synergistic effect of enhanced efflux coupled with reduced influx [93,94]. This coordinated regulation allows bacteria to reduce drug accumulation at low energetic cost.

### 4.2. Porin Repression

Fluoroquinolone molecules enter the periplasmic space of Gram-negative bacteria mainly by passive diffusion through outer membrane porins [94,95]. When porin gene transcription is repressed, porin number decreases or channel properties change, lowering the rate of drug entry and giving efflux pump systems more time to extrude drug that has already entered the cell [94] (Figure 3).

In aquatic pathogenic bacteria, clear evidence supports transcriptional repression of porin genes. Suresh and Pillai [92] analyzed gene expression in *Klebsiella pneumoniae* from freshwater fish farms and reported detection rates of 59.3% for *ompK35* and 62.5% for *ompK36*; these genes frequently co-occurred with AcrAB-TolC efflux pump genes, indicating that altered porin expression and active efflux together form a dual barrier of “reduced influx” and “enhanced efflux.” Tang and Ding [96] conducted transcriptomic analysis of quinolone-resistant mutants of *Vibrio harveyi* and found differential expression of outer membrane protein-related genes, which coincided with changes in genes of the active efflux system and two-component regulatory networks, suggesting that transcriptional regulation of porins is an important component of the bacterial adaptive response to fluoroquinolones.

Comparable regulatory patterns have been reported in other aquaculture-related or analogous contexts. Du et al. [97] examined ciprofloxacin-resistant *Salmonella* isolates from aquatic sources and found downregulation of *ompF* mRNA, along with QRDR mutations such as those in *gyrA*. Akshay et al. [98] monitored gene transcription during resistance induction in *Salmonella* and observed reduced transcription of porin genes including *ompF* and *lamB*. These changes are often linked to transcriptional regulation of efflux pump genes; elevated efflux pump activity can repress porin expression via feedback, further reducing influx efficiency and establishing a positive feedback loop.

Transcriptional repression of porin genes is reversible, not permanent. After drug pressure is removed, porin expression in some strains can return to basal levels [99]. This reversibility enables bacteria to modulate outer membrane permeability as environmental conditions change, balancing the energetic trade-off between resistance and nutrient acquisition.

### 4.3. Plasmid Gene Expression

Plasmid-borne PMQR genes reside on mobile genetic elements, yet they exert resistance functions only when efficiently transcribed and translated in the host. Expression of plasmid-borne genes is regulated at multiple levels.

First, promoter activity of PMQR genes is influenced by the host bacterium’s global regulatory network [73]. The same plasmid may be expressed at different levels in different bacterial species or strain backgrounds; this host dependence partly explains why PMQR genes such as *qnrS* contribute variably to the resistance phenotype across species [80,81]. Second, the copy number of the PMQR-carrying plasmid directly affects gene dosage; resistance genes on high-copy-number plasmids can be expressed at higher levels and thus confer higher resistance to the host [100,101]. However, maintaining plasmids and expressing resistance genes consumes host metabolic resources, creating a trade-off between expression level and fitness. Moreover, when integrons capture PMQR genes, the captured gene cassettes are expressed under the integron’s intrinsic promoter [80]; this regulatory mechanism operates independently of the plasmid’s original regulatory system, further complicating the regulation of PMQR gene expression.

**Figure 4:**
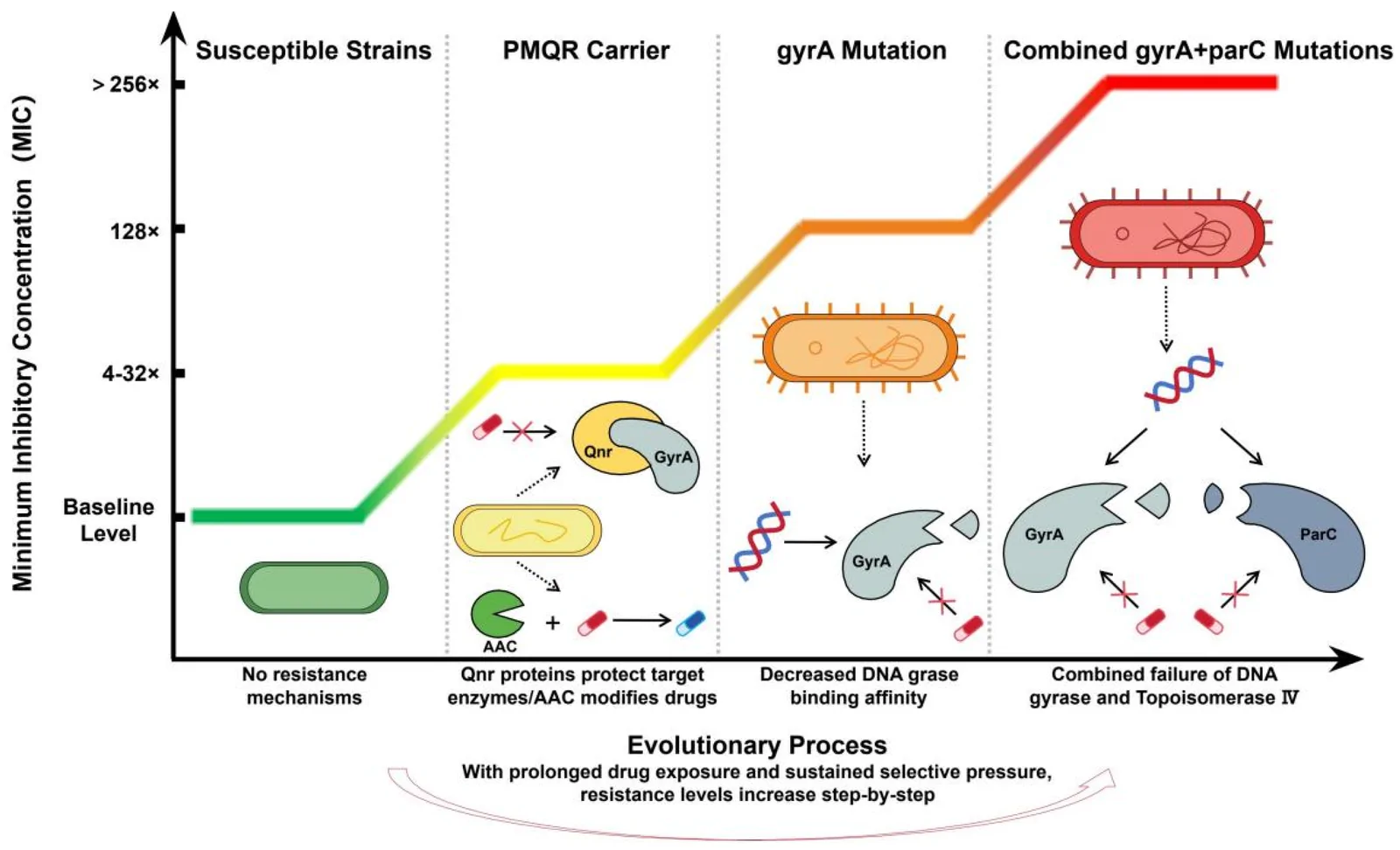
Schematic illustration of one possible pathway of fluoroquinolone resistance accumulation.

PMQR genes confer relatively low-level resistance, typically increasing the minimum inhibitory concentration by 4- to 32-fold [23]. Their stable expression at the transcriptional level nevertheless provides a sufficient survival advantage under sublethal drug concentrations. This low-level resistance enables bacteria to persist in drug-containing environments long enough for the subsequent accumulation of chromosomal target-site mutations (Figure 4) [102]. Transcriptional regulation of PMQR genes, together with the accumulation of mutations in chromosomal genes, drives the transition from low-level to high-level resistance.

## 5. Protein-Level Execution

The gene level encodes resistance information, and the transcriptional level regulates the expression levels of resistance genes; however, resistance functions are ultimately executed by proteins [85,103]. Proteins carry out resistance functions by altering drug targets, protecting targets, extruding drugs, reducing drug entry, or modifying the drug itself [16,25]. These proteins act at drug targets, at drug influx and efflux, or on the drug molecule itself.

### 5.1. Target Alteration and Protection

Fluoroquinolones exert their bactericidal effect by binding to DNA gyrase and topoisomerase IV; the structural state of the target proteins therefore directly determines whether the drug can bind effectively [16,21]. Bacteria respond in two ways: they either alter target protein structure permanently to reduce drug affinity, or transiently protect the targets through accessory proteins to prevent drug binding [74].

Structural alteration of target proteins results from gene mutations within the QRDR [18]. When serine at position 83 of GyrA is replaced by isoleucine, the side-chain polarity and steric properties at this site change, potentially disrupting the water-Mg^2+^ ion bridge and reducing stable fluoroquinolone binding to the target enzyme. Using molecular dynamics simulations, Lessigiarska et al. [104] showed at the atomic level that amino acid substitutions in the QRDR disrupt the dynamic stability of the water-Mg^2+^ ion bridge and increase the binding free energy of the drug-target enzyme complex, thereby shortening the lifetime of the complex and reducing the inhibitory effect of the drug. This mechanism explains why a single GyrA Ser83Ile mutation alone can increase the minimum inhibitory concentration by 4- to 32-fold. When mutations in multiple genes co-occur, both target enzymes lose sensitivity to the drug simultaneously, and the drug can no longer maintain its inhibitory effect through alternative targets, allowing the MIC to increase exponentially [72]. Structural alteration of target proteins is irreversible; once a mutation occurs, the strain and its progeny permanently carry it.

Qnr-mediated protection involves reversible interactions with target enzymes and is mediated by plasmid-encoded proteins [105]. Qnr proteins belong to the pentapeptide repeat protein family and have an elongated structure that enables them to associate with DNA gyrase or topoisomerase IV, sterically blocking fluoroquinolone molecules from approaching the active sites of the target enzymes [25]. Because Qnr proteins do not alter the amino acid sequence of the target enzymes themselves, their protective effect is concentration-dependent: greater plasmid copy number leads to higher Qnr expression and stronger protection [100]. Amin et al. [81] detected a high frequency of *qnrS*-positive strains in *Escherichia coli* from aquatic environments in Bangladesh, in which the ciprofloxacin minimum inhibitory concentration typically increased only 4- to 16-fold, far below the levels observed in strains with target-site mutations. This difference may reflect variations in Qnr expression levels and bacterial genetic backgrounds rather than solely the reversible nature of Qnr-mediated protection. Importantly, the reversibility of Qnr–target interactions does not imply instability of the plasmid-borne *qnr* determinant.

### 5.2. Drug Influx and Efflux

Bacteria can also develop resistance by reducing the intracellular concentration of fluoroquinolones [16,25]. This strategy relies on two classes of proteins: fewer outer membrane porins limit drug entry, while active efflux pumps extrude drug molecules that have already entered the cell [94]. These mechanisms reduce net intracellular drug accumulation by limiting influx and increasing efflux (Figure 3).

Porins are water channel proteins embedded in the outer membrane of Gram-negative bacteria and include OmpF, OmpC, OmpK35, and OmpK36. They provide the main route by which small hydrophilic molecules, including fluoroquinolones, cross the outer membrane [95]. When porin number decreases or channel properties change, drug entry slows markedly. Suresh and Pillai [92] reported that high detection rates of porin genes and genes of the AcrAB-TolC system co-occurred in *Klebsiella pneumoniae* of aquatic origin, indicating that these two mechanisms functionally complement each other: fewer porins reduce influx, whereas enhanced efflux pumps increase efflux, and their combined action markedly lowers net intracellular drug accumulation. Unlike target-site mutations, changes in porin number arise from transcriptional regulation, and the protein-level alteration is reversible [99,106]. Once drug pressure is removed, porin expression can return to basal levels, enabling bacteria to balance resistance with nutrient acquisition.

Active efflux pumps are central to regulating intracellular drug concentration. In Gram-negative bacteria, the tripartite structure of RND family efflux pumps underlies their high efflux efficiency: the inner membrane transporter (e.g., AcrB) recognizes and captures intracellular fluoroquinolone molecules, and the membrane fusion protein (e.g., AcrA) transfers the substrate to the outer membrane channel protein (e.g., TolC), which then extrudes the drug directly out of the cell [87]. The entire process is driven by the proton motive force and does not depend on ATP hydrolysis, allowing sustained operation at relatively low energetic cost [88]. Wu et al. [89], using transcriptome sequencing of induced resistant strains of *Aeromonas schubertii*, observed significantly elevated expression of AcrA and AcrB proteins, and efflux pump inhibitor assays directly confirmed the functional contribution of these protein products, showing a 4-fold reduction in MIC after inhibitor addition. Li et al. [90], through knockout experiments targeting the VmeB protein of *Vibrio parahaemolyticus*, verified efflux pump protein function from another perspective; knockout increased the strain’s susceptibility to enrofloxacin by approximately 5-fold, indicating that VmeB is the primary executor of enrofloxacin efflux in this species. In *Pseudomonas aeruginosa*, the three components of the MexAB-OprM system (MexA, MexB, OprM) together form a complete efflux channel, and Suresh et al. [91] detected these three proteins frequently in strains from freshwater fish sources, further confirming the widespread function of RND efflux pumps in aquatic pathogenic bacteria.

### 5.3. Drug-Modifying Enzymes

The first two classes of proteins act on the drug target and the cell membrane, respectively; the third class acts directly on the fluoroquinolone molecule itself, reducing its antibacterial activity through enzymatic chemical modification [76,107]. AAC(6′)-Ib-cr is currently the best-characterized fluoroquinolone-modifying enzyme in aquatic pathogenic bacteria [108].

AAC(6′)-Ib-cr is a variant of aminoglycoside acetyltransferase with an expanded substrate spectrum [107]. This enzyme N-acetylates the amino group of the piperazine ring in fluoroquinolones such as ciprofloxacin and norfloxacin, thereby reducing their antibacterial activity [25]. Because its activity is restricted to fluoroquinolones bearing a piperazine ring, such as ciprofloxacin and norfloxacin, and is absent against drugs lacking this moiety, such as moxifloxacin, its functional contribution is drug-specific [76]. In aquatic pathogenic bacteria, strains carrying the *aac(6′)-Ib-cr* gene usually show only low-level resistance; however, they are important because they potentiate the resistance phenotype when combined with other mechanisms [76,109].

Relative to target protein mutations and efflux pump overexpression, drug-modifying enzymes make a more limited contribution to fluoroquinolone resistance. This limitation largely reflects the structural features of fluoroquinolone molecules, which make them less susceptible to common modifying enzymes; far fewer enzymes are known to modify fluoroquinolones effectively than to modify aminoglycosides or β-lactams [23,60]. Nonetheless, AAC(6′)-Ib-cr is uniquely valuable because it provides a third resistance pathway independent of target alteration and drug concentration regulation, suggesting that additional enzymatic mechanisms for countering fluoroquinolone pressure may remain to be discovered in bacteria.

## 6. Phenotypic Integration

**Figure 5:**
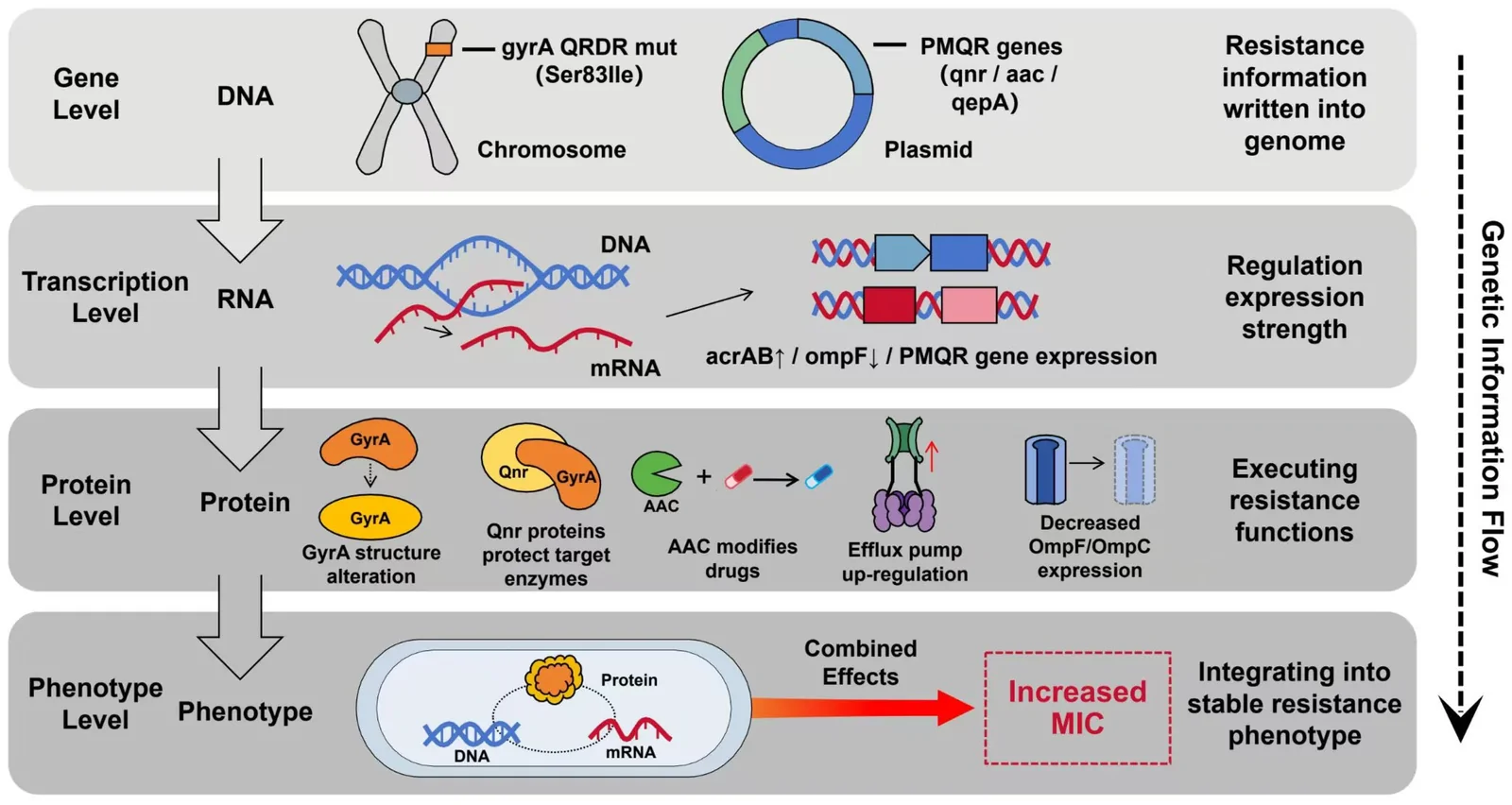
Panoramic integration diagram of the genetic information flow.

Resistance information flows from genes to transcripts and then to proteins. These mechanisms do not act independently; their interactions determine the resistance phenotype. In bacterial cells, they interact and reinforce one another to produce high-level resistance, and this integration yields the resistant phenotype (Figure 5).

### 6.1. Mechanism Synergy

Resistance mechanisms differ in the extent to which they increase the MIC. PMQR genes typically raise the MIC 4- to 32-fold, corresponding to low-level resistance [23]. Single QRDR mutations increase the MIC 4- to 128-fold, corresponding to moderate resistance [21]. In contrast, combined mutations in *gyrA* and *parC*/*parE* can increase the MIC by several hundred-fold or more, representing the decisive step toward high-level resistance [71]. These resistance mechanisms may occur independently or in combination, and their interactions can contribute to different levels of fluoroquinolone resistance (Figure 4) [65,72].

The low-level resistance conferred by PMQR genes initially gives bacteria a survival window under sublethal drug concentrations. Strains carrying *qnr* plasmids can survive early drug exposure, and this survival provides time for the subsequent accumulation of chromosomal QRDR mutations [59]. Because PMQR genes do not alter the amino acid sequence of the target enzymes, bacteria can maintain normal DNA replication under their protection while accumulating additional target-site mutations [82]. Once a *gyrA* mutation arises and confers moderate-level resistance, the low-level protection afforded by PMQR genes combines with the effect of target structural alteration, enabling the strain to tolerate higher drug concentrations [59]. If additional mutations in *parC*/*parE* then emerge, both target enzymes lose susceptibility simultaneously, the drug can no longer act through an alternative target, and the bacteria can shift to a high-level resistance state [110]. This sequential model, proceeding from PMQR priming to *gyrA* mutational transition and then to combined *parC*/*parE* mutations, has been validated experimentally in *Vibrio cholerae* [72] and *Photobacterium damselae* [65].

Efflux pump activation amplifies resistance throughout this process. Whether resistance is introduced through PMQR genes or target-site mutations, the eventual acquisition of a resistant phenotype requires a sufficiently low intracellular drug concentration. Sustained efflux pump activity reduces intracellular drug accumulation, allowing the protective effects of target-site mutations and PMQR to be more fully realized against a background of lower drug concentrations. These mechanisms may have combined effects on fluoroquinolone resistance.

### 6.2. Biofilm Integration

Biofilms are structured bacterial communities that form at solid surfaces or interfaces through processes such as bacterial adhesion, secretion of extracellular polymeric substances, and quorum sensing regulation [93,111]. They do more than impede drug penetration; they also promote population-level tolerance. Unlike heritable resistance, which generally involves genetic changes associated with increased MICs, biofilm-associated tolerance can enhance bacterial survival during antibiotic exposure without necessarily increasing the MIC [30,93].

Biofilms impede drug penetration via extracellular polymeric substances (EPSs) [112,113]. EPSs consist of polysaccharides, proteins, and extracellular DNA and form a dense three-dimensional reticular matrix [114]. As fluoroquinolone molecules traverse this matrix, some drug is adsorbed or bound by charged groups within it, markedly reducing the effective concentration that reaches the membrane surface [115]. Because EPS composition and thickness vary with bacterial species and culture conditions, the extent to which biofilms obstruct drug penetration also differs among biofilm-forming strains of different species [113].

Cells within biofilms also vary metabolically. In mature biofilms, gradients in oxygen, nutrients, and metabolites place bacteria at different depths in distinct metabolic states. Surface-layer cells are metabolically active and susceptible to fluoroquinolones [115], whereas deeper-layer cells have lower metabolic rates, and some enter a dormancy-like state [116]. Because fluoroquinolones act mainly on actively dividing bacteria, these metabolically sluggish deep-layer cells are substantially less susceptible to the drug, enabling them to survive exposure and act as “seeds” for biofilm regrowth [115]. This tolerance stems from metabolic heterogeneity rather than resistance gene mutations and reflects the population-level tolerance conferred by the structural properties of the biofilm.

Biofilms and molecular resistance mechanisms reinforce one another. Li et al. [90] deleted the *vmeB* efflux pump gene in *Vibrio parahaemolyticus* and showed that biofilm-forming ability, motility, and transcription of outer membrane-related genes were all significantly reduced, indicating that the efflux pump protein VmeB not only mediates drug efflux but also indirectly affects biofilm structural integrity by regulating biofilm-related gene expression. Conversely, biofilm formation provides microenvironmental signals that upregulate efflux pump gene transcription, leading to higher efflux pump expression in biofilm-associated bacteria than in planktonic bacteria. This bidirectional regulation forms a self-reinforcing loop at the phenotypic level.

### 6.3. Metabolic Reprogramming

Resistant strains often reprogram metabolism to meet the energy demands of altered targets and sustained efflux [117,118]. Transcriptomic and metabolomic studies have revealed extensive changes in metabolic pathways in resistant strains. In enrofloxacin-induced resistant strains of *Aeromonas schubertii*, Wu et al. [89] found that efflux pump genes were significantly upregulated and that genes of the purine nucleotide biosynthesis pathway (*pur*-series genes) also showed differential expression and became hub nodes in the co-expression network of resistance-associated genes. Changes in purine metabolism may be linked to DNA damage repair and energy metabolism. Under drug pressure, bacteria require more energy to sustain efflux pump operation and DNA repair; because purine nucleotides serve as precursors for ATP and GTP synthesis, shifts in metabolic flux reflect altered energy metabolism [119].

Metabolic reprogramming supports resistance in two ways. First, by adjusting energy metabolic flux, it offsets the additional energy cost of sustained efflux pump activity, allowing bacteria to maintain near-normal growth rates despite carrying multiple resistance mechanisms [118]. Second, metabolic status can alter bacterial drug susceptibility; accumulation or depletion of certain metabolic intermediates may change intracellular pH or redox state and thereby affect the bactericidal efficiency of fluoroquinolones [120]. This metabolic adaptation is not itself a resistance mechanism, but it helps stabilize the phenotype. Table 2 summarizes these mechanisms.

## 7. Conclusions and Perspectives

In this review, we have used the flow of genetic information as a conceptual framework to organize fluoroquinolone resistance mechanisms in aquatic pathogenic bacteria into four interconnected analytical levels: gene, transcription, protein, and phenotype (Figure 5). This framework shows that these mechanisms do not function in isolation but are integrated stepwise along the pathway from DNA to RNA to protein and finally to phenotype, converging into a stable resistance phenotype. Re-examining existing studies through this lens helps clarify where current knowledge remains weak or incomplete.

Three directions merit further study. First, integrated multi-omics studies. Current research on resistance mechanisms in aquatic pathogenic bacteria is largely confined to single-level approaches, mainly genome sequencing and transcriptomic analysis, and integrated analyses of genomic, transcriptomic, proteomic, and metabolomic data remain rare in aquaculture research. Because resistance arises from changes at multiple levels of gene expression, multi-omics approaches are well suited to this task. Cross-level omics analyses of the same strain or population would help clarify the regulatory relationships and causal chain from gene mutation to phenotypic expression, filling current gaps in knowledge between mechanisms at different levels. Second, regulatory functions of non-coding RNAs. In human pathogens, small RNA-mediated post-transcriptional regulation of efflux pump genes such as *acrAB* and porin genes has been relatively well studied; however, the role of non-coding RNAs in fluoroquinolone resistance in aquatic pathogenic bacteria remains largely unexplored. Because non-coding RNA regulation is rapid and reversible, it may be particularly important under sublethal antibiotic exposure. Third, structural biology studies of PMQR proteins. Structural details of Qnr protein binding to DNA gyrase currently derive mainly from data on Enterobacteriaceae; whether PMQR proteins in aquatic pathogenic bacteria show species-specific structural features and functional differences remains unclear. Such data are essential for understanding the molecular basis of the differential contributions of PMQR to resistance across bacterial species.

Beyond conventional resistance mechanisms, DNA damage response and repair pathways also contribute to bacterial survival following fluoroquinolone exposure. Völzing and Brynildsen demonstrated that stationary-phase *Escherichia coli* persisters exposed to ofloxacin require DNA repair during post-treatment recovery, highlighting a survival mechanism that does not necessarily involve increased MICs [121].

Progress in these areas also depends on methodological development. A standardized, comparable antimicrobial resistance surveillance system has not yet been established in aquaculture, and data comparability across studies remains a major issue. At the same time, field validation studies in farming settings remain far fewer than laboratory-based mechanistic studies, and a translational pipeline from laboratory findings to field application has yet to be established. Moreover, the available mechanistic evidence varies in study design, bacterial species, and extent of functional validation. These limitations hinder the translation of mechanistic research into practical control measures and deserve attention.

## Figures and Tables

**Table 1:** Fluoroquinolone Resistance of Common Pathogenic Bacteria from Aquatic Sources.

Region	Isolation Source	Main Pathogenic Bacteria	Antibiotic (Resistance Rate)	References (*n*)	AST Method/Criteria
Jiangsu, China	Diseased fish, shrimp, crabs, etc.	*Aeromonas* spp.	Enrofloxacin 8.58%; Ciprofloxacin 14.87% (2017)/16.75% (2018)	[43] (*n* = 1364)	MIC method (modified)/CLSI
Jiangsu, China	Diseased *Eriocheir sinensis*	*Aeromonas* spp.	Enrofloxacin 4.95%; Flumequine 19.78%	[44] (*n* = 182)	96-well MIC plate method/CLSI 2017
Guangxi, China	Cultured *Crassostrea hongkongensis*	*Vibrio* spp.	Fluoroquinolones (average of 4 agents) 5.1%	[45] (*n* = 64/68/24)	Disk diffusion (K-B)/CLSI M100
Hebei, China	Diseased *Pelodiscus sinensis*	*Aeromonas* spp.	Enrofloxacin 24.3% (2022)/45.6% (2023)/12.5% (2024)	[46] (*n* = 190)	96-well broth microdilution (MIC)/CLSI
Eastern Coastal China (Jiangsu, Fujian, Shanghai)	Diseased *Carassius auratus*, *Channa argus*, etc.	*Aeromonas hydrophila*	Enrofloxacin 30%	[47] (*n* = 20)	Broth microdilution (MIC)/CLSI
Southern Coastal China (Guangdong, Hainan)	Diseased marine fish (grouper, golden pompano, etc.)	*Photobacterium damselae* subsp. *piscicida*	Ciprofloxacin 5.71%; Norfloxacin 5.71%	[48] (*n* = 35)	Disk diffusion (K-B)/CLSI
26 Provinces and Municipalities, China	Retail snails, fish, shrimp, etc.	Non-typhoidal *Salmonella* spp.	Ciprofloxacin 16.8%	[49] (*n* = 728)	Broth microdilution (MIC)/CLSI 2022 (FFC/TGC per CLSI VET01S/EUCAST)
Zhejiang, China	Diseased *Channa argus*	*Aeromonas* spp.	Enrofloxacin 11.11%; Ciprofloxacin 22.22%; Norfloxacin 22.22%	[50] (*n* = 18)	Disk diffusion (K-B)/NCCLS/CLSI
Guangdong, China	Diseased *Micropterus salmoides*	*Edwardsiella piscicida*	Ciprofloxacin 96.25%; Enrofloxacin 40%; Flumequine 16.25%	[51] (*n* = 80)	Broth microdilution (MIC)/CLSI M100 Ed34
Guangdong, China	Retail shrimp and oysters	*Vibrio parahaemolyticus*	Enrofloxacin 1.19%; Ciprofloxacin 4.76%; Norfloxacin 7.14%	[52] (*n* = 84)	Disk diffusion (K-B)/CLSI
Egypt	Tilapia, shrimp, etc.	*Enterococcus* spp.	Ciprofloxacin 74.1%; Levofloxacin 72.2%	[53] (*n* = 54)	Disk diffusion (K-B)/CLSI 2017
Thailand	Diseased freshwater fish (tilapia, bass, etc.)	*Aeromonas* spp. (mainly *Aeromonas veronii*)	Enrofloxacin 21%	[54] (*n* = 86)	Disk diffusion (K-B)/CLSI VET04
Bangladesh	Shrimp and aquaculture environment (water, sediment)	*Vibrio* spp. (*Vibrio cholerae*, *Vibrio parahaemolyticus*, *Vibrio alginolyticus*)	Ciprofloxacin 15.7%; Levofloxacin 17.7%	[55] (*n* = 51)	Disk diffusion (K-B)/CLSI
Pakistan	Freshwater fish aquaculture water and sediment	Mixed bacteria (*Pseudomonas aeruginosa*, *Achromobacter xylosoxidans*)	Norfloxacin 30%; Enrofloxacin 27%; Ciprofloxacin 18%	[56] (*n* = 101)	Disk diffusion (K–B); CLSI where applicable, with adaptations for environmental isolates
Iran	Fish, shrimp, lobster	*Acinetobacter baumannii*	Ciprofloxacin 45%	[57] (*n* = 20)	Disk diffusion (K-B)/CLSI
United States	Retail shrimp	*Enterococcus* spp.	Ciprofloxacin 84.55%	[58] (*n* = 110)	Broth microdilution (MIC)/CLSI/NARMS/FDA breakpoints

Note: Sample sizes refer to the number of bacterial isolates subjected to antimicrobial susceptibility testing (AST). Testing methods and interpretive criteria vary across studies and may limit direct comparisons of reported resistance rates. MIC, minimum inhibitory concentration.

**Table 2:** Fluoroquinolone Resistance Mechanisms in Aquatic Pathogenic Bacteria.

Mechanism Category	Information Level	Molecular Event	Involved Genes/Proteins	Reported MIC Fold Change	Representative Bacterial Species	References
Target-site mutation (single)	Gene level + Protein level	QRDR point mutations alter the structure of target enzymes	GyrA Ser83Ile/Asp87Asn	4–128×	*Aeromonas hydrophila*, *Vibrio parahaemolyticus*, *Aeromonas veronii*	[61,62,67]
Target-site mutation (combined)	Gene level + Protein level	Simultaneous mutations in two target sites	GyrA Ser83Ile + ParC Ser85Phe	>256×	*Photobacterium damselae*, *Vibrio cholerae*	[65,72]
PMQR (target protection)	Gene level + Protein level	Qnr proteins bind target enzymes and block drug binding	*qnrS*/*qnrVC*	4–32×	*Vibrio cholerae*, *Aeromonas* spp., *Escherichia coli*	[79,80,81]
PMQR (drug modification)	Gene level + Protein level	Reduced antibacterial activity via *N*-acetylation	*aac(6′)-Ib-cr*	4–16×	*Escherichia coli* (aquatic environment)	[25,81]
PMQR (efflux)	Gene level + Protein level	Active drug extrusion by plasmid-encoded efflux pumps	*qepA*/*oqxAB*	4–16×	*Escherichia coli* (aquatic environment)	[25,81]
Efflux pump overexpression	Transcriptional level + Protein level	Upregulated expression of efflux pump proteins	AcrAB-TolC/VmeAB/MexAB-OprM	4–32×	*Aeromonas schubertii*, *Vibrio parahaemolyticus*, *Pseudomonas aeruginosa*	[89,90,91]
Reduced membrane permeability	Transcriptional level + Protein level	Downregulated porin expression decreases drug influx	OmpF/OmpK35/36	2–8×	*Klebsiella pneumoniae*, *Salmonella* spp.	[92,97]

Note: MIC fold changes vary with bacterial species, genetic backgrounds, and experimental conditions. Corresponding references are provided for each mechanism. MIC, minimum inhibitory concentration.

## Data Availability

No new data were created or analyzed in this study. Data sharing is not applicable to this article.
